# Supplementary material for: HIVprotI: an integrated web based platform for prediction and design of HIV proteins inhibitors
Source: J Cheminform. 2018 Mar 9;10:12. doi: 10.1186/s13321-018-0266-y (PMC5845081; doi:10.1186/s13321-018-0266-y)
Supplement: Supplementary file 2 — Additional file 2. Source code of HIVProtI web server. [file 13321_2018_266_MOESM2_ESM.zip › HIVprotI_Source-code/hivprotI/jsme/JSME_depict_smiles.html]

# JSME in depict mode initialized with a SMILES

SMILES conversion and 2D coordinates computation are performed by the Open Source Chemistry Library OpenChemLib


### Taxol

Width changes when the browser window changes, the molecule is rescaled to fit if needed
